# Management of people with epilepsy during the COVID-19 pandemic: a national survey among epileptologists in China

**DOI:** 10.1186/s42494-020-00030-0

**Published:** 2020-10-27

**Authors:** Zhenxu Xiao, Ding Ding, Shichuo Li, Zhen Hong

**Affiliations:** 1 Institute of Neurology, Huashan Hospital, Fudan University, Shanghai, 200040 China; 2 China Association Against Epilepsy, Beijing, China

**Keywords:** COVID-19, Epilepsy, Survey, Consensus, Opinion, China

## Abstract

**Background:**

Compared to the healthy people, people with comorbid medical conditions are more vulnerable in the context of the coronavirus disease 2019 (COVID-19) pandemic, including the people with epilepsy. Besides a consensus recommendation by multi-national epilepsy specialists, the situation of the epilepsy management during the pandemic has seldom been reported.

**Methods:**

The China Association Against Epilepsy carried out an online nationwide survey among its board members in April 2020. One hundred and thirty board members from 22 provinces, 5 autonomous regions, and 4 municipalities across China responded to the questionnaires. They reported the situation of clinical practice and gave opinions on the management of people with epilepsy between January 13th and March 31st, 2020, a time period concentrated with confirmed COVID-19 cases.

**Results:**

The proportions of patients consulting through telephone or online (88.4%) and of patients with regular case review (93.9%) were highest in the high-risk area, as reported by the responders. The patients in the high-risk area were more likely to have increased episodes of seizures (17.7%), aggravated psychological disorders (30.2%), and less accessibility to anti-seizure medications (ASMs) (77.2%). Regular ASMs supply (74.6%), medical consultation (69.2%), and psychological aids (29.2%) were urgently needed for people with epilepsy.

**Conclusions:**

This study demonstrated the most common dilemma faced by people with epilepsy in policy circumstances during the COVID-19 epidemic in China. The opinions raised by Chinese epileptologists may provide reference for epilepsy care in other countries.

**Supplementary information:**

**Supplementary information** accompanies this paper at 10.1186/s42494-020-00030-0.

## Background

On March 12, 2020, the coronavirus disease 2019 (COVID-19) was declared as a pandemic by the World Health Organization (WHO) [1]. As a response to the COVID-19 outbreak, China implemented strict quarantine measures in most communities and villages, and imposed a city lockdown in Wuhan. Compared to the healthy people, people with comorbid medical conditions are more vulnerable in the context of this public health issue, including the people with epilepsy. A consensus recommendation by multi-national epilepsy specialists suggests that people with epilepsy receive medical care at home rather than in health care facilities, and take measures to reduce the possibility of seizure exacerbation by adhering to the medications [2]. The impact of the COVID-19 pandemic on people with epilepsy in Italy [3] and Arabia [4] has been reported recently. A Chinese study has revealed severe psychological distress among people with epilepsy during this crisis [5]. However, on the other aspect, the management of people with epilepsy during the pandemic in China has not been reported.

In April 2020, the China Association Against Epilepsy (CAAE) conducted a nationwide retrospective survey among epileptologists, aiming to assess the situation of management of people with epilepsy during the COVID-19 epidemic in China. In this article, we report the results of the survey and comment on the major problems and challenges of epilepsy management during the COVID-19 epidemic from the perspective of Chinese epileptologists.

## Materials and methods

### Survey participants

The CAAE is a national non-government organization dedicated to the treatment, prevention, and control of epilepsy in China. It is a non-profit association of neurologists, neurosurgeons, pediatric neurologists, and other experts serving epilepsy care nationwide. All board members of the CAAE are epileptologists with a MD degree from second-grade class-A or third-grade class-A hospitals. Two hundred and thirty-five board members of the CAAE were eligible survey participants.

### Questionnaire

On April 3, 2020, the CAAE sent a two-part questionnaire to all the board members by email. The first part of the questionnaire quantitatively assessed the clinical practice situation in case management of people with epilepsy between January 13 and March 31, 2020, when the number of confirmed COVID-19 cases rose from 41 to 81 554 in China [6, 7]. The survey questions to each board member included: (1) the number of patients for consultation (including clinical consultation and other forms of teleconsultation); (2) the proportion of telephone/internet-based consultation; (3) the proportions of new presentations and regular case reviews (follow-ups) in all patients (including face-to-face clinic patients and telemedical patients); (4) the proportions of patients with increased episodes of seizures, with status epilepticus, and with aggravated psychological disorders, among the regular case reviews; (5) the proportion of patients who should but did not come for regular case review; and (6) the proportion of patients who were unable to obtain anti-seizure medications (ASMs). In the second part, 5 questions were designed to qualitatively elicit opinions from each board member: (1) What are the causes for seizure aggravation or increased frequency of seizure in patients for case review? (2) What are the reasons for the aggravation of psychological disorders (e.g. anxiety or depression) in patients for case review? (3) Why didn’t the patients come for regular case review albeit they should? (4) Why were the patients unable to obtain ASMs? (5) What are the urgent needs for people with epilepsy in this COVID-19 epidemic?

### Statistical analysis

Three groups of geographic area were defined according to the accumulating number of confirmed COVID-19 cases by April 20, 2020: the high-risk area identified for Hubei Province due to the highest number of cases, the low-risk areas identified for 15 provinces with < 300 confirmed cases, and the moderate-risk areas for the other 15 provinces. The continuous variables are presented as median and range, while the categorical variables are presented as number and frequency (%). Continuous variables with abnormal distribution were analyzed with the Kruskal-Wallis test, and the categorical variables were analyzed with the Pearson chi-square test and Fisher’s exact test when applicable.

## Results

As the high-risk area, Hubei Province had the largest number of COVID-19 cases (*n* = 68 100), and the highest gross domestic product (GDP) compared with the low- and moderate-risk areas. However, there was no significant difference in the proportion of the urban population, the number of beds and practitioners, and the epilepsy prevalence among the three levels of epidemic areas (Table A.1).

One hundred and thirty CAAE board members from 22 provinces, 5 autonomous regions, and 4 municipalities completed the questionnaires. Among them, 3 were from the high-risk area, 89 were from the moderate-risk areas, and 38 were from the low-risk areas. Table 1 showed the characteristics of the people with epilepsy reported by the CAAE board members during the survey. The high-risk area had the highest proportions of patients consulting through telephone or online manners (88.4%) and of patients with regular case review (93.9%). Among the patients with case review, those in the high-risk area were more likely to have increased episodes of seizures (17.7%) and aggravated psychological disorders (30.2%), such as anxiety and depression. In addition, 60.2% of the patients in the high-risk area should but did not come for regular case review. A higher proportion of patients (77.2%) in the high-risk area was unable to obtain ASMs, compared to those in low- and moderate-risk areas.

**Table 1:** Characteristics of people with epilepsy reported by the CAAE board members during the survey

Variates	All	COVID-19 epidemic severity areas			
		Low-risk area	Moderate-risk area	High-risk area	*P* value
Number of surveyed responders, *n*	130	38	89	3	
Average number of patients with consultation reported per responder, median (range)	155 (6, 1 400)	115 (18, 1 400)	179 (6, 1 210)	200 (150, 766)	
Telephone- or internet-based consultation^a^, *n* (%)	81 (32.3)	93 (38.8)	67 (26.9)	329 (88.4)	< 0.001
Patients with regular case review^a^, *n* (%)	172 (68.6)	159 (66.5)	171 (68.2)	349 (93.9)	< 0.001
Patients with increased episodes of seizures^b^, *n* (%)	26 (15.4)	26 (16.3)	25 (14.9)	62 (17.7)	0.006
Patients with status epilepticus^b^, *n* (%)	2 (0.9)	1 (0.8)	2 (1.0)	3 (0.9)	0.184
Patients with aggravation of psychological disorders^b^, *n* (%)	32 (18.8)	42 (26.2)	26 (15.1)	106 (30.2)	< 0.001
Patients who did not come for regular case review albeit they should^c^, *n* (%)	460 (73.3)	724 (82.0)	345 (67.6)	527 (60.2)	< 0.001
Patients who were unable to obtain ASMs^a^, *n* (%)	88 (35.0)	94 (39.4)	78 (31.1)	287 (77.2)	< 0.001

*ASMs* antiseizure medications

^a^Among all the patients with consultation; ^b^Among patients with regular case review; ^c^Among all patients who should come for regular case review; *n *(%), the average number of patients reported per responder (%)

Table 2 shows the three most common answers for each subjective question. According to the answers of 130 responders, withdrawal, decreased dosage, alternative ASMs, and ASMs shortage were among the leading causes of aggravation or increased frequency of seizure (81.5%), while lifestyle changes (26.9%) and psychological disorders, such as anxiety and fear (18.5%) were also reasons for poor disease management. The major causes for the aggravation of psychological disorders were ASMs shortage (54.6%), fear of suffering from COVID-19 (40.8%), and irregular lifestyle (14.6%). For patients who should but did not come for regular case review, quarantine and traffic restrictions (73.8%), fear of suffering from COVID-19 (33.8%), and the epidemic of COVID-19 (15.4%) were major obstacles. Breakdown of ASMs supply systems (51.5%), quarantine and traffic restrictions (47.7%), and suspension of clinical services (6.9%) were the main causes of ASMs inaccessibility. As suggested by the responders, maintaining regular ASMs supply (74.6%), providing consultation, especially ASMs treatment instruction (69.2%), and psychological aids (29.2%) were the most urgent needs for people with epilepsy in the COVID-19 epidemic.

**Table 2:** The top three answers for each qualitative question

Items	Responders
	(*N* = 130)
What are the causes of seizure aggravation or increased frequency in patients for case review?	
Withdrawal, decreased dosage, alternative ASMs, and ASMs shortage, *n* (%)	106 (81.5)
Lifestyle change, *n* (%)	35 (26.9)
Psychological disorders (anxiety and fear), *n* (%)	24 (18.5)
What are the reasons for the aggravation of psychological disorders (e.g. anxiety or depression) in patients for case review?	
ASMs unavailability; ASMs shortage, *n* (%)	71 (54.6)
Fear of suffering from COVID-19, *n* (%)	53 (40.8)
Irregular lifestyle, *n* (%)	19 (14.6)
Why didn’t the patients come for regular case review albeit they should?	
Quarantine and the traffic restrictions, *n* (%)	96 (73.8)
Fear of suffering from COVID-19, *n* (%)	44 (33.8)
Epidemic of COVID-19, *n* (%)	20 (15.4)
Why were the patients unable to obtain ASMs?	
Breakdown of ASMs supply systems, *n* (%)	67 (51.5)
Quarantine and traffic restrictions, *n* (%)	62 (47.7)
Suspension and limitations of clinical services, *n* (%)	9 (6.9)
What are the urgent needs for people with epilepsy in this COVID-19 crisis?	
Maintaining regular ASMs supply, *n* (%)	97 (74.6)
Providing consultation, especially ASMs treatment instruction, *n* (%)	90 (69.2)
Psychological counsultation, *n* (%)	38 (29.2)

*ASMs* antiseizure medications, *COVID-19* coronavirus disease 2019

## Discussion

In this nationwide survey, we found that people with epilepsy from the low-, moderate- and high-risk areas had significant differences in approach to seeking medical consultation, psychological conditions, and ASMs accessibility. Regular ASMs supply, medical consultation, and psychological aids were the most urgent demands of them in the COVID-19 epidemic in China. To our knowledge, this is one of the few expert consensuses focusing on people with epilepsy during the COVID-19 pandemic.

**Fig. 1:**
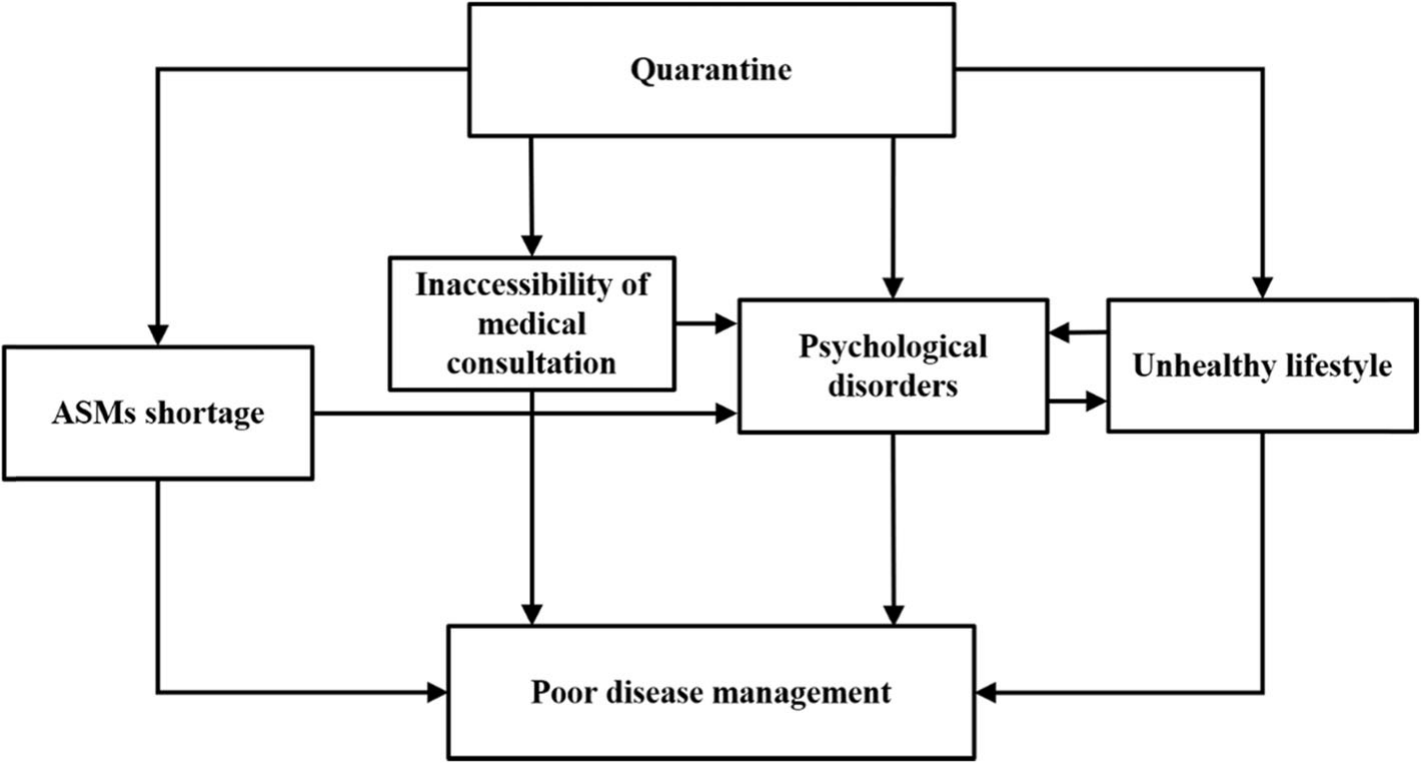
Summary of the most-frequently suggested impacting factors on epilepsy care during the COVID-19 pandemic. ASMs, antiseizure medications

The consensus on the impacting factors for epilepsy care during the COVID-19 epidemic (Fig. 1) may explain the poor management of epilepsy in China, reflected by the finding that patients in the high-risk area were more likely to have increased episodes of seizure and aggravation of psychological disorders (Table 1). According to the views of the epileptologists, the availability of ASMs seems to have played a crucial role in epilepsy management. Withdrawal or dose change would impact the efficacy of ASMs and cause the failure of seizure control. On the other hand, ASMs shortage would cause stress and anxiety for people with epilepsy, which may potentially cause aggravation of seizure. Therefore, the medication delivery systems should be optimized and maintained, and doctors should provide a long-time prescription of ASMs for convenience. A recent-published multi-national expert consensus also emphasized the importance of ASMs adherence and supply during the COVID-19 pandemic [2]. The percentages of patients with worsening of seizures/increased seizure frequency were 18% and 29.5% in Italy and Saudi Arabia, respectively [3, 4], which were higher than our result. Since these studies had different data collection methods, the discrepancy should be interpreted with caution.

Quite a few people with epilepsy need medical consultation during the lockdown period, especially for those who had increased episodes of seizure or status epilepticus. The difficulty to obtain professional medical advice from epileptologists would also arouse the anxious emotion, which could cause worse disease management. Under the nationwide strict lockdown and quarantine measures in China, telemedicine played an important role in delivering medical services for people with epilepsy. Online or telephone-based consultations could avoid person-to-person contact, and were extremely convenient and helpful in the high-risk area [2]. Therefore, we recommend people with epilepsy take full advantage of the telemedicine resources.

Psychological disorders, including anxiety, depression, panic, and stress, are common among people with epilepsy (18.8%). Many modifiable and unmodifiable factors can contribute to the mental health issue. A study in southwest China has concluded that the psychological distress is highly prevalent among people with epilepsy (13%) during the COVID-19 epidemic in China [5]. However, in a questionnaire-based research project in Saudi Arabia, 59.4% of people with epilepsy reported increased stress [4]. The different definitions of psychological stress in these studies may explain the diverse results.

An unhealthy lifestyle is another factor for aggravation of seizure, e.g. being addicted to electronic products and television, alcoholism, lacking physical exercises, and an irregular diet. Thus, psychological aids should also include the education of a healthy lifestyle for people with epilepsy.

This survey investigated the situation of people with epilepsy between January and March, the early-to-mid stage of the epidemic in China. Through the efforts of the whole nation, China has already come through the severe situation. However, many countries are still experiencing the hardest time. The results of the survey may provide reference with respect to the management of people with epilepsy during the peak stage of the COVID-19 pandemic. Empirically, the epileptologists were likely to be more skillful to tackle problems during the later stage of the epidemic. Future studies need to focus on the evaluation of strategies by epileptologists in the later period of this global crisis.

The current study has several limitations. First, the response rate in the high-risk and moderate-risk areas are 100% and 98%. Only 38% of board members responded to the survey in low-risk areas, and this may cause a selection bias. Second, our survey contained subjective answers only based on the responders’ estimation. For example, the psychological status of people with epilepsy was mostly evaluated by clinicians through their own clinical experience instead of the standardized diagnosis procedure. The face-to-face clinical evaluation of patients was hardly achievable because of the high risk of COVID-19 infection in the hospital. The clinician-based observation would underestimate the prevalence of depression and anxiety among people with epilepsy, which indicates that our survey results may be conservative. Third, the economic status, medical resource, and epilepsy prevalence in different areas were potential confounders in this survey. Compared to other areas, we did not find a significantly lower GDP, number of beds and clinicians, proportion of the urban population, or significantly higher epilepsy prevalence in Hubei. We recognized that there may be other potentially relevant factors that have not been taken into account. Fourth, all the questions in the questionnaire should be designed as open-ended or multi-perspective questions to avoid inductivity. Fifth, the board members of CAAE are likely to be top experts of epilepsy in China. Therefore, our study might have neglected opinions from epileptologists other than CAAE board members. Some of them are working in less developed areas and may have met with different situations in clinical practice. Finally, we only received questionnaires from 3 responders in the high-risk area Hubei Pfrovince. This small sample size made the survey less representative and may cause a selection bias. The non-significant difference of some characteristics, e.g. patients with status epilepticus among three epidemic severity areas might also due to the limited sample size. Further larger sampled studies are needed to verify our results.

## Conclusion

This study illuminated the most common dilemma faced by people with epilepsy in policy circumstances during the COVID-19 epidemic in China. The opinions raised by Chinese epileptologists may provide reference for epilepsy care in other countries. Further studies on the physical and psychological health of people with epilepsy in this public health crisis are needed.

## Supplementary information

**Additional file 1: Table A.1.** Characteristics of COVID-19 epidemic areas with different severity in 31 provinces.

## Data Availability

The datasets used and/or analyzed during the current study are available from the corresponding author on reasonable request.

## Abbreviations

ASMs: Anti-seizure medications
CAAE: China Association Against Epilepsy
COVID-19: Coronavirus disease 2019
GDP: Gross domestic product
WHO: World Health Organization
