# Supplementary material for: Management of people with epilepsy during the COVID-19 pandemic: a national survey among epileptologists in China
Source: Acta Epileptol. 2020 Oct 27;2:19. doi: 10.1186/s42494-020-00030-0 (PMC7588342; doi:10.1186/s42494-020-00030-0)
Supplement: Supplementary file 1 — Additional file 1: Table A.1. Characteristics of COVID-19 epidemic areas with different severity in 31 provinces. [file 42494_2020_30_MOESM1_ESM.docx]

| **Table A.1. Characteristics of COVID-19 epidemic areas with different severity in 31 provinces** | | | | | |
| --- | --- | --- | --- | --- | --- |
|  |  | COVID-19 epidemic severity areas | | |  |
|  |  | Low-risk area | Moderate-risk area | High-risk area |  |
|  | All (31 provinces) | (15 provinces) | (15 provinces) | (Hubei province) | *P value* |
| Number of confirmed COVID-19 cases^a^, *n* [min, max] | 328 [1, 68 100] | 147 [1, 256] | 787 [328, 1 580] | 68 100 | <0.001 |
| Gross Domestic Product (GDP)^b^, billion RMB [min, max] | 2 480 [170, 10 800] | 1 410 [170, 2 580] | 3 980 [1 360, 10 800] | 4 580 | <0.001 |
| Percentage of urban population^c^, % | 36.89 | 35.41 | 37.29 | 40.21 | 0.676 |
| Beds/1 000 people^e^, n [min, max] | 2.35 [1.52, 6.30] | 2.62 [1.52, 4.14] | 2.17 [1.78, 6.30] | 2.14 | 0.367 |
| Medical practitioners/1 000 people^f^, n [min, max] | 1.50 [0.960, 4.20] | 1.73 [0.960, 2.69] | 1.45 [1.00, 4.20] | 1.50 | 0.356 |
| Epilepsy prevalence^g^, % [min, max] | 1.60 [0.290, 8.30] | 1.55 [0.570, 8.30] | 1.94 [0.290, 6.96] | 1.70 | 0.750 |

Notes:

a. Confirmed COVID-19 cases in China by April 20, 2020, available at https://k.sina.com.cn/article_3164957712_bca56c10040018cdy.html?from=news&subch=onews

b. Provincial GDP ranking in China, 2019. Available at https://www.sohu.com/a/371574758_611449

c. Urban and Rural Populations (10 000 people) in China, 2000. *Health Statistics Yearbook of China P.342*.

d. The number of medical institutions by regions in China, 2004. *Health Statistics Yearbook of China P.10*.

e. Beds in hospitals and health centers per 1 000 people in China. *Health Statistics Yearbook of China P.68*.

f. The number of medical practitioners per 1 000 people in all regions in China, 2004. *Health Statistics Yearbook of China P.28*.

g. Gu L, Liang B, Chen Q, Long J, Xie J, Wu G, et al. Prevalence of epilepsy in the People's Republic of China: A systematic review. *Epilepsy Res* 2013; 105, 195-205.
